# Supplementary material for: Identification of photoperiod-induced specific miRNAs in the adrenal glands of Sunite sheep (Ovis aries)
Source: Front Vet Sci. 2022 Jul 22;9:888207. doi: 10.3389/fvets.2022.888207 (PMC9354845; doi:10.3389/fvets.2022.888207)
Supplement: Supplementary file 1 [file Data_Sheet_1.ZIP › Supplementary file/miRDeep2 file.zip!/miRDeep2 file/Know_Structure/LP42A2/oar-mir-655.pdf]

miRBase precursor : oar-mir-655
Total read count : 2110
oar-miR-655-5p read count : 3
oar-miR-655-3p read count : 2106
remaining reads : 1

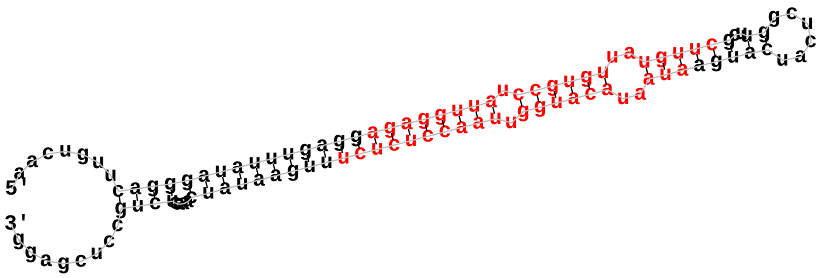

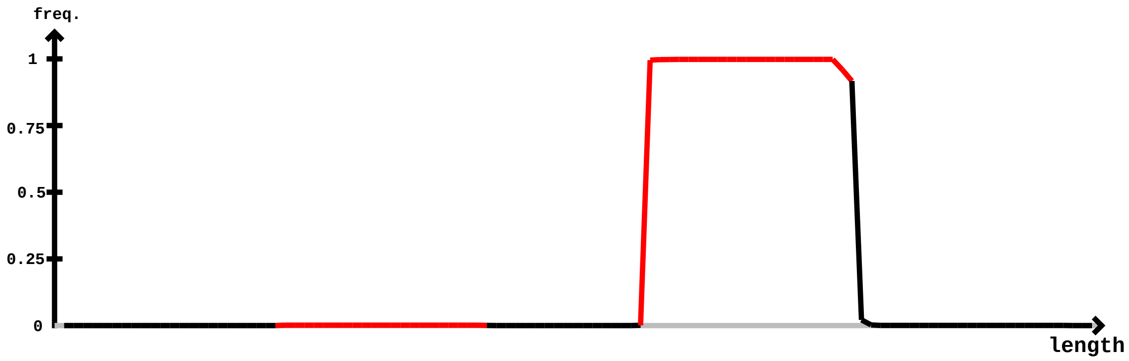

oar-miR-655-5p

oar-miR-655-3p

```
5'- aacuguucagggauauuugaggagagguuauccguguuauguucgcuuggcucaucaugaauaauacaugguuaaccucucuuugaauaucaaacucugccucgagg   -3'    exp
    ...........(((((((((((((((((((.((((((..((((((..((......))))))))..)))))).)))))))))))))))))))...(((......))).    reads      mm        sample
    ......................agagguuauccguguuauguu................................................................    2          0         seq
    ......................agagguuauccguguuauguuc...............................................................    1          0         seq
    ...........................................................aauaauacaugguuaaccucu...........................    2          0         seq
    ............................................................auaauacaugguuaaccucu...........................    79         0         seq
    ............................................................auaauacaugguuaaccucuc..........................    89         0         seq
    ............................................................auaauacaugguuaaccucucu.........................    1886       0         seq
    ............................................................auaauacaugguuaaccucucuu........................    40         0         seq
    ............................................................auaauacaugguuaaccucucuuu.......................    4          0         seq
    .............................................................uaauacaugguuaaccucucu.........................    4          0         seq
    ..............................................................aauacaugguuaaccucucu.........................    1          0         seq
    ...............................................................auacaugguuaaccucuc..........................    1          0         seq
    ..................................................................................uugaauaucaaacucugccucg...    1          0         seq
```
